# 

**Published:** 1857-08

**Authors:** 


					﻿Receipts on Account of Medical Journal, by J. C. Hughes.
Dr. Allen JB, a $2 00
Blakeslee, 2 00
Buckner S W, 2 00
Benedict A G, 2 00
Carpenter II, 2 00
Daniels AW, 2 00
Davidson J W 200
Dean A, 2 00
Barley, J II, 2 00
Farrington S, 2 00
Dr. Fleming J, $2 00
Hamilton, F, 4 00
Harper, ó M 2 00
Hudson, A T, 2 00
Hendershott, 4 00
Matthews, J J 2 00
.McPherson G, 2 00
Mason,	1	00
Putnam II, 2 00
Rauch J I’,	4	00
Dr. Skinner, $2 00
Siveter, Thos, 4 0<)
Staples, Jos 2 Oo
Slonaker, 2 Od
Thompson, F 2 00
Turner J C.	4	00
Tidrick, L M. 2 00
Van Patten N, 2 00
West, C, 2 00
We would call attention to the cards, &c., to be
found upon our advertising sheet. Keokuk, it will be seen,
offers large inducements to physicians who dispense their
own drugs, as also druggists, to make their purchases here,
and thereby save the time and expense incident to a visit to
the East for that purpose. We have no hesitation, from our
personal knowledge of the men engaged in the business here,
as also from examination of many of their articles, in saying
that physicians and druggists may be accommodated, by or-
dering or purchase in person, with every thing in the line,
with the certainty of being pleased with the character and
address of our dealers, as also with their purchases.
Within a few months Messrs. Alley & Hayden have open-
ed a house (wholesale and retail) for the sale of all kinds of
Rubber goods, and what is of more interest to our subscri-
bers, they keep on hand all of the improved gutta percha
and rubber appliances for surgical and medical use. See ad-
vertisement.
Among the advertisements from St. Louis, Mo., we would
call especial attention to that of M. W. Alexander & Co.;
for, from some business intercourse with this house, we feel
free to say that what they promise to do, that they perform.
To those going or sending to St. Louis for the purpose of
purchasing chemicals, drugs, surgical instruments, &c., we
would say give them a call, and you will find no cause of re-
gret for so doing.	w. r. m.
We would call the attention of our subscribers who
have remitted subscriptions to the second page of our cover,
where they will find acknowledgment of all receipts since
last issue, except a few which, too late for this number, will
be acknowledged in our next.
The new building for our State Medical University.
65 by 90 feet, is pushing forward to an early completion.
				

